# Controls on Gut Phosphatisation: The Trilobites from the Weeks Formation Lagerstätte **(**Cambrian; Utah**)**

**DOI:** 10.1371/journal.pone.0032934

**Published:** 2012-03-14

**Authors:** Rudy Lerosey-Aubril, Thomas A. Hegna, Carlo Kier, Enrico Bonino, Jörg Habersetzer, Matthieu Carré

**Affiliations:** 1 Department of Palaeontology and Historical Geology, Senckenberg Research Institute, Frankfurt am Main, Germany; 2 Department of Geology and Geophysics, Yale University, New Haven, Connecticut, United States of America; 3 Department of Geology, Western Illinois University, Macomb, Illinois, United States of America; 4 Back to the Past Museum, Puerto Morelos, Quintana Roo, Mexico; 5 Department of Palaeoanthropology and Messel Research, Senckenberg Research Institute, Frankfurt am Main, Germany; 6 Institute of Evolutionary Sciences, University Montpellier II, Montpellier, France; Institut de Biologia Evolutiva - Universitat Pompeu Fabra, Spain

## Abstract

Despite being internal organs, digestive structures are frequently preserved in Cambrian Lagerstätten. However, the reasons for their fossilisation and their biological implications remain to be thoroughly explored. This is particularly true with arthropods – typically the most diverse fossilised organisms in Cambrian ecosystems – where digestive structures represent an as-yet underexploited alternative to appendage morphology for inferences on their biology. Here we describe the phosphatised digestive structures of three trilobite species from the Cambrian Weeks Formation Lagerstätte (Utah). Their exquisite, three-dimensional preservation reveals unique details on trilobite internal anatomy, such as the position of the mouth and the absence of a differentiated crop. In addition, the presence of paired pygidial organs of an unknown function is reported for the first time. This exceptional material enables exploration of the relationships between gut phosphatisation and the biology of organisms. Indeed, soft-tissue preservation is unusual in these fossils as it is restricted to the digestive structures, which indicates that the gut played a central role in its own phosphatisation. We hypothesize that the gut provided a microenvironment where special conditions could develop and harboured a source of phosphorus. The fact that gut phosphatization has almost exclusively been observed in arthropods could be explained by their uncommon ability to store ions (including phosphorous) in their digestive tissues. However, in some specimens from the Weeks Formation, the phosphatisation extends to the entire digestive system, suggesting that trilobites might have had some biological particularities not observed in modern arthropods. We speculate that one of them might have been an increased capacity for ion storage in the gut tissues, related to the moulting of their heavily-mineralised carapace.

## Introduction

### Background

The Cambrian explosion was a major episode of biological diversification, which was associated with the development of complex marine ecosystems [1]. During this ecological revolution, the diversification of feeding strategies no doubt played a major role, but the importance of this diversification remains difficult to assess. Indeed, the unusual morphology of Cambrian metazoans prevents easy comparisons to modern analogues and rarely provides unambiguous clues about their feeding habits. This is particularly true with arthropods, typically the most diverse fossilised organisms in Cambrian ecosystems [2], [3], which commonly lack the kind of appendage specialization that characterises feeding strategies in modern arthropods. Fossilised digestive structures represent an as-yet underexploited alternative to appendage morphology for inferences on the biology of primitive arthropods [2], [4]. Despite being internal organs, their fossilisation is unusually common in Cambrian Lagerstätten. However, the reasons for this preservation and the biological implications remain to be thoroughly explored. Here we describe phosphatised digestive structures of three species of trilobites from the Middle Cambrian Weeks Formation Lagerstätte (Utah). Their exquisite, three-dimensional preservation allows unique details on trilobite internal anatomy to be revealed. In addition, similarly-preserved enigmatic structures under the pygidium, probably representing as-yet unknown organs, are reported for the first time. The unusual preservation of the digestive structures is thoroughly explored, in an attempt to reveal anatomical and systematic controls on gut fossilisation. Exploring the possible relationships between the anatomy and function of the gut and its preservation may permit novel inferences on aspects of Cambrian metazoan biology which are otherwise inaccessible.

### Geological setting

**Figure 1:**
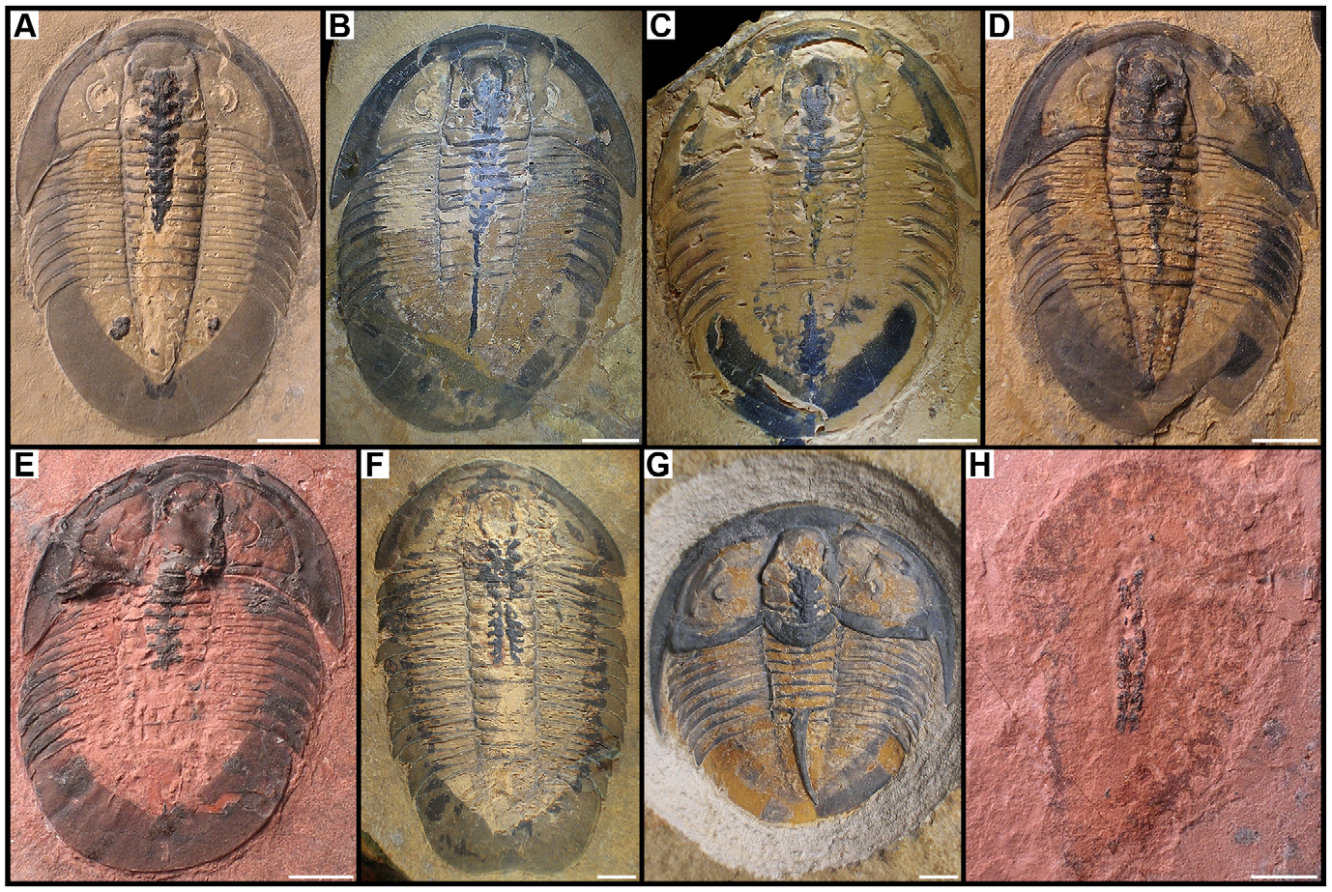
Arthropods bearing phosphatised digestive structures from the Middle Cambrian Weeks Formation. All specimens are complete and preserved dorsal side up, with head to top of image. A–E, *Meniscopsia beebei*. A, BPM 1017. B, BPM 1000. C, BPM 1001. D, BPM 1020. E, BPM 1018. F, *Coosella kieri*, BPM 1002. G, *Geneviella granulatus*, UU 11071.01. H, undetermined arthropod, BPM 1019. Scale bars: 5 mm.

The House Range of central Utah is home to three Cambrian Konservat-Lagerstätten: the Marjum Formation, the Weeks Formation, and the Wheeler Shale. The Weeks Formation has received the least scientific attention of the three while paradoxically being well-known to amateur palaeontologists for its well-preserved and complete trilobites. The unit is a 300 m thick sequence of thin-bedded lime mudstones, locally enriched in siliciclastic sediments [5]. It is interpreted as a shallowing-upward sequence, transitional between the outer-shelf shale and lime mudstones of the underlying Marjum Formation and the shallow subtidal carbonates of the overlying Big Horse Limestone Member of the Orr Formation. Boundaries between the three units are conformable. A Guzhangian age (Cambrian, Series 3) can be proposed for most, if not the entirety of the Weeks Formation [5]. Laurentian Cambrian Lagerstätten were all deposited on the inner portion of the outer shelf [3], which likely explains the important similarities of their faunas. In this regard, the Weeks formation may be of particular interest, since it probably represents a shallower palaeoenvironment. However, the most remarkable feature of the Weeks Formation Lagerstätte is the quality of arthropod digestive system preservation. Digestive structures belonging to diverse arthropods are preserved in three dimensions (Figures 1A–H) and are best exemplified by the trilobites, which are discussed below.

## Results

### Mode of preservation

**Figure 2:**
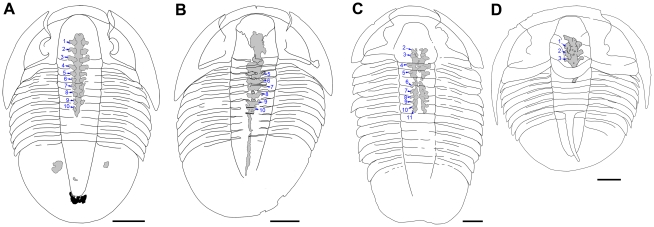
Interpretative sketches of some specimens shown in Figure 1. A, *Meniscopsia* beebei, BPM 1017; dark stain behind the posterior tip of pygidial axis may represent material exuded from the anus after the death. B, *M. beebei*, BPM 1000. C, *Coosella kieri*, BPM 1002. D, *Geneviella granulatus*, UU 11071.01. Pairs of digestive caeca are numbered from front to rear. Scale bars: 5 mm.

**Figure 3:**
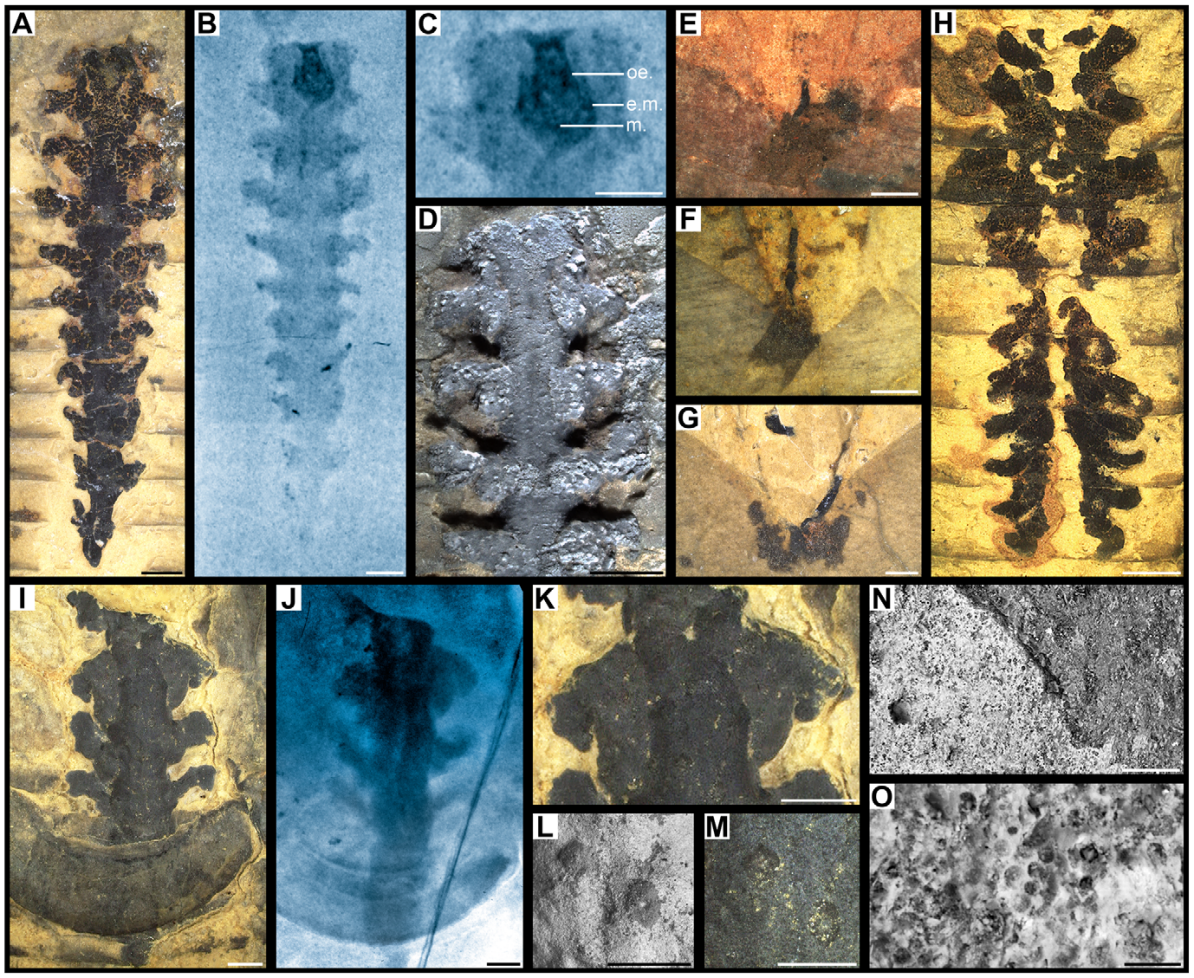
Phosphatised digestive structures of trilobites from the Middle Cambrian Weeks Formation. A–D, G, *Meniscopsia beebei*, BPM 1017. A, anterior half of the digestive system, specimen in alcohol. B, same as (A), microradiograph. C, detail of the anterior portion of digestive system, microradiograph; ventral structures interpreted as the oesophagus (oe.) surrounded posteriorly by material exuded (e.m.) from mouth (m.) after death. D, cephalic portion of digestive system, specimen coated with MgO. G, posterior tip of pygidial axis, specimen in alcohol. E, *M. beebei*, BPM 1018, posterior tip of pygidial axis, specimen in alcohol. F, *M. beebei*, BPM 1020, posterior tip of pygidial axis, specimen in alcohol. H, *Coosella kieri*, BPM 1002, large portion of digestive system, specimen in alcohol. I–M, O, *Geneviella granulatus*, UU 11071.01. I, anterior, mostly cephalic portion of digestive system, specimen in alcohol. J, same as (I), microradiograph. K, anteriormost portion of digestive system, specimen in alcohol. L, two silica grains within phosphatised digestive tract, Scanning electron microscope (SEM) image. Q, same as (P), specimen immersed in alcohol. O, detail of the surface of phosphatic material, SEM image. N, *M. beebei*, BPM 1000, textures of phosphatised digestive caeca (left) and neighbouring cuticle (right), SEM image. Scale bars: 2 mm for (H), 1 mm for (A–G, I–K), 0.5 mm for (L, M), 50 µm for (N), and 10 µm for (O).

Three-dimensionally preserved digestive structures (DS) can be observed in three different species of ptychopariid trilobites (Figures 1A–G, 2A–D, 3A–O)— *Meniscopsia beebei* [6], *Coosella kieri* [6], and *Genevievella granulatus* [7]—as well as in several non-trilobite arthropods (e.g. Figure 1H). In all the specimens with fossilised DS, most of the dorsal exoskeleton is very thin or absent, allowing the dark, often bluish DS to be observed from the dorsal side of the specimens (Figures 1A–G). EDX analyses on several specimens (*M. beebei* and *C. kieri*) have revealed that the DS are composed predominantly of C, O, Ca, P and Si (with minor amounts of Mg, Fe, and Al). F has also been repeatedly detected, but not in all the samples (Table S1). These data suggest that the DS contain a notable amount of calcium phosphate (abbreviated CaPO_4_ hereafter for simplicity), along with calcium carbonate and silica. In both the cuticle and the surrounding matrix, neither P nor F could be detected with confidence using EDX. However, mass spectrometry analyses of the matrix surrounding one specimen has revealed P at an extremely low concentration (<300 ppm; Table S2). SEM investigations suggest that CaPO_4_ precipitated within the digestive lumen, with no evidence of phosphatised digestive tissues. The phosphatic material exhibits a spongy texture in surface owing to the presence of tiny (*c*. 1–4 µm) spherical impressions (Figures 3N, O). In *G. granulatus* (Figures 3L, M), represented by a single specimen in this study, there are a few crystalline inclusions of about 500 µm that are chiefly composed of Si and O (with minor amounts of Ca, P, and Br), as revealed by EDX analyses. We therefore interpret them as clastic grains similar to those observed in the matrix, albeit rarely. In addition to DS, one specimen of *M. beebei* (BPM 1017) displays a pair of enigmatic structures below the pygidial pleurae (Figure 1A). Their microscopic appearance suggests the same mode of preservation as the DS (i.e. phosphatisation).

### Description of the digestive structures

**Figure 4:**
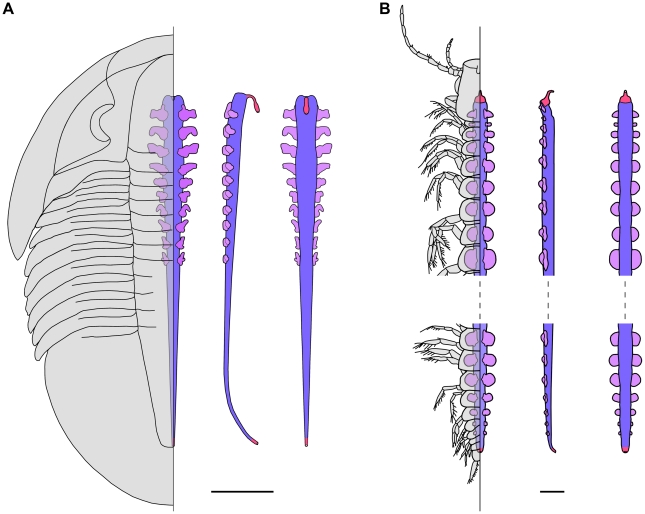
Reconstructions of the digestive systems of a trilobite and a remipedian. A, digestive system of the trilobite *Meniscopsia beebei* in dorsal, right lateral, and ventral views (from left to right). B, digestive system of the remipedian *Speleonectes gironensis* in dorsal, right lateral, and ventral views (from left to right; reconstruction based on [65], [66]). In both reconstructions, the foregut and hindgut are in bright pink, the midgut tract in blue-violet, and the midgut caeca/glands in lavender. Scale bars: 5 mm for (A) and 1 mm for (B).

The outstanding quality of the preservation allows previously unrecorded details of the digestive system of trilobites to be observed [8] (for detailed descriptions of each specimen, see Text S1). This is particularly true for *M. beebei*, where DS are known from seven specimens, which permits a complete description of the species's digestive system (Figure 4A). It is composed of a central tract and three cephalic and seven thoracic digestive caeca (Dc; Figures 1A, 2A, B, 3A, B). Anteriorly, the tract bears a short but broad sagittal depression, giving a bilobate appearance (Figures 2A, D). Uniformly wide (*c*. 25% of the maximum width of the glabella [transverse, tr.]) beneath the glabella, it tapers posteriorly until the seventh thoracic segment (*c*. a half of cephalic width), and then remains roughly constant in width in its posterior portion (Figures 1A, B). The paired Dc are similar in shape but decrease in size posteriorly. Their insertions occur dorso-laterally on the digestive tract and they are wide (exsagittally, exs.), especially those of the first four pairs (Figure 3A). The anterior halves (exs.) of the Dc project abaxially and slightly anteriorly for a distance equal to half the width (tr.) of the tract. The best-preserved specimen (BPM 1017) displays a shallow sagittal furrow dorsally along the caeca-bearing portion of the tract (Figure 3D), which might have hosted the dorsal heart. On the posterior portion of the tract (e.g. BPM 1000), fine, grossly transversal furrows are visible. Three specimens exhibit a dark stain behind the posterior tip of the pygidial axis (Figures 3E–G), which may represent material exuded from the anus after death. In addition, one specimen (BPM 1017) possesses a pair of rounded structures under the pygidial pleurae (Figure 1A). Microradiograph of specimen BPM 1017 shows ventral structures beneath the anteriormost portion of the dorsal tract (Figures 3B, C). These are tentatively interpreted as the oesophagus and posteriorly-facing mouth, with the surrounding material probably exuded from the mouth after death. This observation confirms the presence of a J-shaped gut in trilobites. The mouth is just in front of the first of pair of Dc, which corresponds to a short distance behind the anterior wings of the hypostome, which are visible in *in situ* position.

The single specimen of *G. granulatus* available for study exhibits well-preserved DS anteriorly (Figures 1G, 2D, 3I). Its tract is similar to that of *M. beebei*, except it is more inflated dorsally, lacks a sagittal furrow, and contains rare silica grains (Figures 3L, M). Three pairs of Dc are visible, strongly decreasing in size posteriorly. They are similar to the Dc of *M. beebei* in gross morphology, size, and orientation. Only the Dc of the first pair differs slightly from those of *M. beebi* by having particularly large bases (exs.) and projecting laterally with a strong posterior curve (Figure 3K). Unfortunately, microradiographs of the specimen provide no evidence of an underlying oesophagus (Figure 3J).

Phosphatised DS could also be observed in one specimen of *C. kieri* under the posterior third (sagittally, sag.) of the glabella and the anterior half (sag.) of the thorax (Figures 1F, 3H). They are broken (tr.) at the third thoracic axial ring, which allows recognition of an anterior portion and a posterior portion with four and six pairs of Dc respectively. The posterior portion has apparently contracted along the antero-posterior axis, as suggested by the slight posterior displacement of the Dc under the third and fourth thoracic axial rings and the presence of two pairs of Dc under the fifth and sixth thoracic axial rings. As in *M. beebei*, the Dc arise dorso-laterally from the tract, but they differ from all other known trilobite Dc by projecting postero-laterally.

## Discussion

### Organisation of trilobite digestive system

The preservation of portions of the trilobite digestive system is particularly rare. As recently reviewed by Lerosey-Aubril *et al*. [8], purported remains of digestive structures have been described and/or figured in about twenty species of trilobites, but usually from one, rarely more specimens. In most cases, this preservation is limited to some structures (e.g. Dc *vs.* digestive tract) and is incomplete. The morphology of the cephalic digestive structures remains completely unknown in most species and poorly understood in the others [8]. In addition, different modes of preservation can be involved (e.g. dark markings on the exoskeleton/internal mould, mineral infillings, cavities/external moulds), making the comparisons between different specimens even more difficult. However, from all these data has emerged the hypothesis that two, possibly three general types of digestive systems existed in trilobites [8], [9]. One consists of a simple, posteriorly-tapered tract, which was flanked laterally by metamerically-paired digestive caeca in the head and anterior part of the thorax. A second type, consisting of a crop (*sensu* Lerosey-Aubril *et al.* [8], i.e. a pouch-like structure under the glabella) followed posteriorly by a tract devoid of digestive caeca, might have occurred in trilobites exhibiting a well-developed frontal lobe of the glabella [8]. Lastly, as indirectly inferred from impressions on the dorsal exoskeleton [10]–[12], some agnostids might have possessed a digestive system in which some digestive caeca were modified into large and ramified diverticulae. The digestive systems described herein are all of the first type. Their delicate, 3D preservation allows unprecedently detailed observations to be made. This, in turn, offers a good opportunity to test different hypotheses concerning critical aspects of trilobite digestive system organisation. For instance, specimens of *G. granulatus* and *M. beebei* confirm that in this type of digestive system (i.e. type 1 described above), there is no abrupt enlargement (tr.) of the anterior portion of the digestive tract evocative of the differentiation of a crop [8]. Indeed, although notably wider (tr.) anteriorly than posteriorly, the digestive tract gradually tapers rearwards. Specimens of *M. beebei* also confirm that trilobites lacked digestive caeca in the posterior part of the trunk (Figure 1B) [8]. This observation is noteworthy since posterior digestive caeca occur in organisms like naraoids [2], which are generally considered close to trilobites both phylogenetically and ecologically. However, only three pairs of cephalic digestive caeca occur in *M. beebei* and *G. granulatus*, and not four as recently hypothesised [8]. The specimens from the Weeks Formation also contribute to the debate concerning the position of the mouth relative to the hypostome in trilobites [13], [14]. If our interpretation of the microradiograph of BPM 1017 is correct (Figures 3B, C), the mouth is only slightly more posterior than the anterior wings in *M. beebei*. This suggests that the posterior part of the hypostome might have constituted the floor of a preoral cavity where the gnathobases of the post-antennal cephalic appendages processed food before ingestion [13], [15].

The Dc of trilobites have frequently been compared to the digestive glands of modern arthropods and more specifically, arachnids and remipedian crustaceans [4], [9], [16]. This has even led some authors to infer predatory habits for them [4], [17]. Chelicerates possess a greater number of digestive glands compared to most crustaceans, making them a more attractive comparison for trilobites. However, these glands are large and complex structures, which frequently have different functions and exhibit different shapes depending on their location within the body (e.g. [18], [19]). Therefore, it does not seem appropriate to compare this morphologically and functionally complex digestive system of modern chelicerates to the simple structures observed in trilobites (not to mention the well-differentiated sucking-stomach possessed by many chelicerates). In contrast, with its posteriorly-tapered tract bearing numerous small, homonymous, and metamerically-paired outpocketings (‘digestive glands’) [20], the digestive system of remipedians can be regarded as the only reasonable functional analogue among modern arthropods to the trilobite digestive system described herein (Figures 4A, B). This apparent ‘primitive’ nature of the remipedian gut makes this comparison all the more interesting. The remipedian digestive system has a simple foregut [21] and lacks differentiation of the epithelial cells in the midgut [20]; both observations demonstrate that it is far less complex than the digestive systems of other crustaceans. Predation by remipedians on other crustaceans or even small fish has been repeatedly recorded [20]–[23], but recent observations of specimens of *Speleonectes* kept in aquaria have suggested these organisms may also engage in filter-feeding [24]. This indicates that the gross organisation of the gut of remipedians and of the trilobites described herein is not unambiguously correlated with predatory habits. For instance, deposit feeding [14] could explain the presence of silica grains in the gut of *G. granulatus*. However, the absence of such grains in the gut of other specimens and the significant texture and composition differences between the gut filling and matrix indicate that direct sediment ingestion was not a major part of its feeding strategy. In our opinion, the grains are better interpreted as having been incidentally ingested in the course of normal feeding. In summary, the morphology of the digestive structures of trilobites does not provide clear indications about their feeding habits, but future investigations on the functioning of the digestive system of remipedians could prove critical in this regard.

### Gut phosphatisation and its relationships to trilobite biology

The unique preservation of the digestive structures of the trilobites from the Weeks Formation gives new insights into trilobite biology. Phosphatisation is a well-known mode of preservation for soft tissues [25]–[31], but its role in the fossilisation of invertebrate gut contents has rarely been investigated (see [4] for an exception to this). Partial phosphatisation of the gut has been observed in arthropod decay experiments [32], [33], but neither the nature of the mineralised material (gut tissue *vs*. gut content), nor controlling factors (e.g. P content of diet, stage of moult cycle) were examined.

A quiet environment, free from scavengers, is a prerequisite for the preservation of soft tissues, but phosphatisation also necessitates a confined microenvironment, acidic conditions, a source of P, and some bacterial activity [34]. Soft-tissue preservation in the Weeks Formation trilobites is restricted to DS alone, indicating that the chemical conditions favourable to the formation of CaPO_4_ were confined to the closed chemical system of the gut after death. Normally in marine environments, the precipitation of calcium carbonate (CaCO_3_) is favoured over CaPO_4_ [25], with CaPO_4_ favoured in acidic environments. The presence of a small quantity of material exuded from the anus in several specimens (or possibly the mouth in BPM 1017; Figures 3C, E–G) demonstrates that exchange with the external environment occurred, but was limited, probably due to oral and anal sphincters. The isolation of this microenvironment after death could only have lasted until the gut lost its integrity. Experiments on modern crustaceans have shown that the gut is particularly prone to decay in most circumstances [32], [33], suggesting that precipitation of CaPO_4_ in the trilobite gut likely occurred soon after death. In addition, the digestive lumen was almost certainly acidic [32], due to the release of acidic byproducts by microbes metabolising the gut contents or the gut tissue itself after death [28], favouring CaPO_4_ precipitation. Fossilised microbes have already been reported associated with phosphatised digestive glands in a Cambrian arthropod [35], but none were observed on our material using the SEM; however, their original presence is suggested by the spongy texture of the phosphatic material (Figures 3N, O), indicating possible microbial moulds [26], [31], [36]. Lastly, the extensive phosphatisation of the trilobite DS required a relatively large amount of P to be present in the gut. The extremely low P content in the surrounding sediment (Table S2) and the closed-system nature of the gut argue strongly for an internal origin for this element. We identified two potentially important internal sources of P: the digestive tissues and the gut contents.

In many arthropods, the epithelial cells of the midgut or the midgut glands contain mineral concretions (‘spherites’) that are especially rich in Ca and P (e.g. in chelicerates [18], [19], [37]–[39], crustaceans [40]–[45], insects [46], [47]). These spherites are thought to be involved in various vital processes (e.g. excretion [45], [46], detoxification [41], [48], and storage of ions [43], [46]). They are usually released into the digestive lumen [42], or dissolved intracellularly to permit the remobilisation of their elements [43], [45]. Similar spherites have also been observed in the midgut tissues of non-arthropod ecdysozoans (e.g. tardigrades [50], onychophorans [51], and nematodes [52]); consequently, it seems highly probable that they occurred in primitive arthropods like trilobites. The storage of CaPO_4_ spherites has already been proposed as an explanation of the DS phosphatisation in *Leanchoilia superlata* and other arthropods from the Burgess Shale [4], [53], [54]. Rare non-trilobite arthropods from the Kaili [35] and Emu Bay lagerstätten [55] and from the Weeks Formation (Figure 1G) similarly have their Dc or posterior part of the tract preserved as CaPO_4_. In all these cases, the spatial pattern of gut phosphatisation is consistent with an origin of the P from CaPO_4_ spherites stored in the midgut glands or, as in modern horseshoe crabs [18], released in their lumen and transiently stored in the posterior portion of the tract (e.g. in *Sidneyia* [56]). How the ions of these spherites were transferred from the gut epithelium and mobilised after death for the phosphatisation of the gut contents is difficult to ascertain. We can only speculate that the mechanism involved might have been similar to that recently proposed to explain the phosphatisation of the mid-dermis of fossil frogs, as it implies the recruitment of P and Ca ions initially stored as granules [34]. However, the phosphatisation of the gut in the trilobites from the Weeks Formation (particularly in *M. beebei*) is not restricted to the Dc or the posterior portion of the digestive tract, but concerns virtually any portions of the digestive system from the mouth to the anus (Figures 1A–E, 2A, B, 3B, C). This has no equivalent in other Cambrian arthropods, which suggests that trilobites might have had some biological or ecological particularities. One possibility is that the Ca and P-rich spherites might have been stored in the tissues of the entire midgut (not only the Dc), thus providing a more substantial and widespread source of P. In few modern arthropods, spherites have been observed in the epithelium of the midgut tract [19], [46], and it is therefore possible that in trilobites, spherite storage was not restricted to the Dc. Alternatively, trilobites might have merely stored a greater volume of spherites in the epithelium of their Dc. Such an increase in storage capacity would not be surprising given that midgut CaPO_4_ spherites are thought to play an important role in the storage of Ca ions in extant arthropods [43], [46] and that trilobites had probably a great need for Ca due to their heavily calcified dorsal exoskeleton. This exoskeleton was shed and replaced many times during their life [57]. As a consequence, trilobites likely needed a greater capacity for Ca storage, most probably in the form of CaPO_4_ as in the digestive glands of modern crustaceans [43], to facilitate a rapid hardening of the new cuticle after each moult. Ion storage in the digestive glands of modern crustaceans is at its peak immediately prior to moulting [43], [58] and therefore, gut phosphatisation may have only occurred in trilobites that died at this point in their moult cycle. In this regard, it is noteworthy that all the specimens with fossilized digestive structures investigated herein exhibit unusually thin dorsal exoskeletons, as if they had been affected by a partial resorption of the ions they contained (especially Ca). If this interpretation is correct, these specimens would indeed represent individuals which died just prior moulting. This relationship between moult cycle and time of death may explain the rarity of preserved trilobite guts not only in the Weeks Formation, but also in other exceptionally preserved biotas.

The organic matter present in the gut might have represented an alternative or complementary source of P, as in the phosphatisation of vertebrate gut contents [34]. In the trilobites from the Weeks Formation, the unusually extensive phosphatisation of the digestive system may have been aided by the ingestion of food particularly rich in P, allowing this element to occur in relatively high concentrations throughout the gut. In doing so, P-rich food would have not only provided the necessary P ions, but also facilitated the precipitation of CaPO_4_ in the entire digestive system in diminishing the significance of other factors (e.g. a low pH) critical to it. Small (< 5mm), undetermined inarticulate brachiopods are found in great abundance in the horizons that have yielded complete polymerid trilobites in the Weeks Formation. The organophosphatic shells of these minute brachiopods could be a potential source of P ions. However, SEM and x-ray investigations have not permitted identification of the remains of organisms in the fossilized guts, but it seems unlikely that once crushed by trilobite gnathobases, the small and weakly mineralized shells of these inarticulate brachiopods would have left recognizable pieces in their gut, especially after their ions have been remobilized to permit the post-mortem precipitation of CaPO_4_. Microbial mats might also have represented a potentially P-rich, semi-fluid food for trilobites, since they are known to concentrate P in modern environments [59]. However, no fossilized microbial mats have as-yet been reported in the Weeks Formation, and the low concentration of P in the sediment surrounding the trilobites argues against the presence of such mats in these horizons [59].These are just two examples aiming to show that our present data do not necessarily imply that trilobites were microphagous. However, a precise determination of their diet (if possible) would require further analyses (e.g. thin sections or acid dissolutions of the phosphatic material) that we are not permitted to perform on the existing material.

### The enigmatic pygidial organs

**Figure 5:**
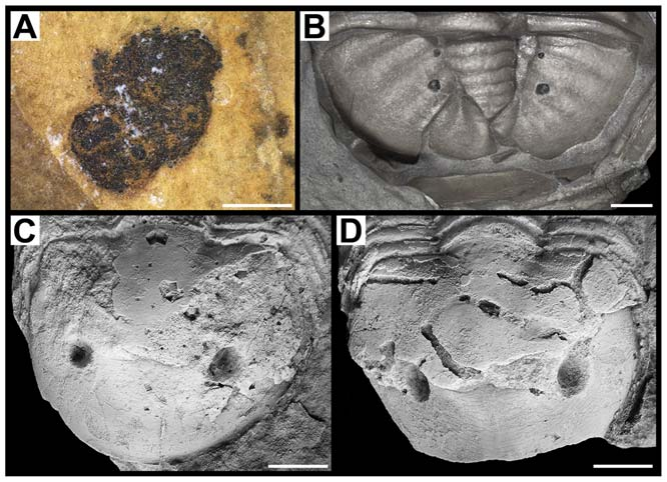
Enigmatic paired structures under the pygidium of trilobites. A, *Meniscopsia beebei*, Weeks Formation (Utah), Middle Cambrian, BPM 1017, enigmatic structure under the left pleural field of the pygidium, specimen immersed in alcohol. B, *Phacops rana*, Ledyard Shale Formation (New York), Middle Devonian, SMF 92660, pygidium displaying two pairs of ovoid structures protruding from the above lying, locally eroded cuticle. C, D, *Ectillaenus* sp., exact locality unknown (Morocco), Ordovician. C, SMF 87908, internal mould of pygidium showing two large pits. D, SMF 87911, internal mould of pygidium showing two large pits. Scale bars: 1 cm for (C, D) and 1 mm for (A, B).

The paired structures under the pygidium of one specimen of *M. beebei* exhibit preservation much like that of its digestive system (Figures 1A, 5A), suggesting they might have had comparable internal conditions at the time of death. Some modern arthropods possess paired structures in the posterior region of their body that are known to be sites of storage for CaPO_4_ spherites. These modern examples include the malpighian tubules of some insects (e.g. *Musca domestica* [60]) or the modified digestive diverticulae in some crustaceans (e.g. *Orchestia cavimana* [61]). However, these organs are connected to the midgut from which they derive embryologically, while the paired structures observed in this trilobite appear physically separated from the digestive tract (Figures 1A, 5A). They are even separated from one another, preventing comparisons with other paired organs (e.g. reproductive organs) that at least in some arthropods could have had a similar position in the body. Also, we do not see any obvious equivalents in modern arthropods and we can only speculate that these structures might have represented transient storage centres for Ca (and P), like the sternal plates of extant terrestrial isopods [62].

Two pairs of such structures could be observed under the pygidium of a specimen of *Phacops rana* [63] from the Middle Devonian Ledyard Shale Formation (New York; Figure 5B). In addition, large pits on two internal pygidial moulds of *Ectillaenus* sp. (Figures 5C, D) and altered areas on the pygidial cuticle of a specimen of *Asaphellus catamarcensis* [64] confirm that similar pygidial organs may have occurred in various trilobites. The unexpected discovery of such structures suggests that the internal anatomy of trilobites was probably more complex than generally appreciated.

### Conclusions

The study of the trilobites from the Weeks Formation emphasises some of the unique factors involved in gut phosphatisation. The digestive system itself plays a dual role: housing a favourable microenvironment, and providing a source of P (i.e. digestive tissues or gut contents). Gut phosphatisation is, therefore, closely tied to the biology of organisms. Because of this, new inferences on physiological or ecological aspects of extinct animals can be made. For instance, the fact that gut phosphatisation has almost exclusively been observed in fossil arthropods suggests that they, like their modern representatives and unlike many other metazoan groups, commonly store ions (especially Ca and P) in their digestive tissues. Likewise, the unusually extensive phosphatisation of the gut of trilobites might indicate an increased capacity for the storage of Ca and P ions in the digestive tissues, possibly in relation to the greater amount of Ca ions needed at each moult for the hardening of their heavily-mineralised carapace. DS are the most frequent internal structures in Cambrian Lagerstätten, which suggests that the digestive system may also have a role in its preservation through other processes than phosphatisation. Investigating these other forms of gut preservation might shed light to critical aspects of the biology of Cambrian metazoans.

## Materials and Methods

Nine trilobites and five undetermined arthropods from the Weeks Formation, each with phosphatised digestive structures, were available for study. In addition, we figure herein three specimens exhibiting remains of pygidial organs. These specimens are housed in the palaeontological collections of the Back to the Past Museum (BPM), the Senckenberg Research Institute (SMF), and the University of Utah (UU). The single specimen from the collections of the University of Utah was given on loan for study to RL-A by the curator of this collection, Quintin Sahratian. M. Sahratian gave his authorization for all the analyses performed on this specimen (SEM, EDX, x-ray) and his permission to illustrate the specimen in our contribution.

The specimens were photographed using a digital camera Leica DFC420 mounted on microscope Leica MZ12.5. For some pictures, the specimens had been immersed under diluted ethanol to facilitate the observations of details. Some pictures of BPM 1020 have been taken after the specimen had been coated with MgO. Six of the seven trilobites from the Weeks Formation have been investigated using scanning electron microscopes (SEM) with attached energy dispersive X-ray (EDX) systems. BPM 1000, 1002, 1018, and 1020 have been studied at the University Montpellier II (Table S1; SEM FEI QUANTA-FEG 200, EDX module Oxford Instruments X-max, 15 kV), BPM 1001 at Yale University (SEM FEI XL-30 ESEM-FEG, EDX Princeton Gamma Tech, 30 kV), and UU 11071.01 and a thin section of the matrix of BPM 1001 at the Senckenberg Research Institute (SEM JEOL JSM-6490LV, EDX EDAX-Ametek, 20 kV). All samples were uncoated and analysed under low vacuum. Homogenized powdered samples of the matrix of BPM 1001 were dissolved using HF and HNO_3_ acids during 48 hours, then 1000 fold diluted with high purity water, and finally enriched with 1ppb Indium (used as an internal calibration standard) before being analysed twice using an inductively coupled plasma mass spectrometer (ICP–MS) AGILENT 7700X (Table S2). All the trilobite specimens from the Weeks Formation were x-rayed using a microfocus tube (Feinfocus FXE 110–52, max. resolution 5 µm) and for screening, the enlarged image was projected on a 5 megapixel digital x-ray flat panel sensor (Hamamatsu C7984CK12,) with a genuine resolution of 50 µm. Live images and 12–20 times integrated stored images were studied on the computer monitor and the settings of the x-ray tube voltage were optimized between 40kV and 100kV. Subsequently, 70 megapixel ultra-high resolution microradiographs were obtained for specimens BPM 1020 and UU 11071.01 by long-term exposure (180–300 sec) of x-ray storage screens with a genuine resolution of 25 µm, which were processed with a laser scanner (Dürr HD–CR 35 NDT). Resulting pictures were processed (noise reduction, gamma correction, sharpening) using Photoshop CS2.

## Supporting Information

Text S1 **Detailed descriptions of the specimens**.
(DOC)

Table S1 Energy Dispersive X-ray (EDX) analyses of the digestive structures, cuticle, and matrix in four trilobite specimens from the Middle Cambrian Weeks Formation. *Meniscopsia beebei* (BPM 1000, BPM 1018, BPM 1020) and *Coosella kieri* (BPM 1002). Composition is expressed in atomic percentage (%) and the analysed area in square micrometres (µm^2^). Some elements were not detectable on spectra and therefore were not considered (NC) in the composition estimations. Though recognized on spectra, other elements had estimated proportions below the equipment's lower limit of reliability (0.3%). The digestive structures are characterized by the abundance of P and significantly higher proportion of Ca. The cuticle exhibits the highest proportion of Mg, which is possibly linked to its original composition. The matrix is distinguished from the other two materials by its higher concentration of Al and (to a lesser extent) Fe, as well as the presence of K just above the reliability threshold. The presence of Si in all three materials is probably linked to secondary silicification of the cuticle and (to a lesser extent) the digestive structures, whereas the presence of Si in the matrix is likely the result of aluminosilicate minerals.
(DOC)

Table S2 Mass spectrometry analyses of the matrix of BPM 1001 (*Meniscopsia beebei*) from the Middle Cambrian Weeks Formation. Concentrations are expressed in parts per million (ppm). Note the extremely low value for P.
(DOC)
