# Supplementary material for: Controls on Gut Phosphatisation: The Trilobites from the Weeks Formation Lagerstätte (Cambrian; Utah)
Source: PLoS One. 2012 Mar 14;7(3):e32934. doi: 10.1371/journal.pone.0032934 (PMC3303877; doi:10.1371/journal.pone.0032934)
Supplement: Text S1 — Detailed descriptions of the specimens. (DOC) [file pone.0032934.s001.doc]

The specimens are housed at the Back to the Past Museum (BPM) and the Department of Geology and Geophysics of the University of Utah (UU).

*Meniscopsia beebi* Robison & Babcock, 2011

BPM 1000. Digestive structures occur under the posterior two-thirds of the glabella and almost the entire post-cephalic axis. Only the posteriormost portion of it is missing because it is crossed by a glued area which has been needed due to a breakage of the slab. The portion of the digestive system extending from the glabella to the axial ring of thoracic segment 7 is strongly weathered and it is not always easy, especially anteriorly, to distinguish the digestive caeca from the tract. All in all there seems to be 10 pairs, 3 cephalic ones and 7 thoracic ones. The most anterior portion of the digestive tract has been smashed within the anterior lobe of the hypostome. There is a clear trend toward a reduction of digestive caeca from front to rear. The posterior portion of the tract is narrower (tr.), especially near the posterior tip, and it seems to have been more or less filled with ingesta when the animal died, which more or less protrudes dorsally. Its surface bears numerous, thin, sub-transversal furrows.

BPM 1001. The digestive system is visible under the posterior two-thirds of the glabella and the axial rings of all the thoracic segments but the most posterior one. It reappears under the anterior part of the pygidial axis, but it is progressively replaced posteriorly by a broad mass of material amalgamated with the dorsal cuticle, probably because of a breakage of the wall of the tract. The anterior portion of the digestive system up to thoracic segment 3 is weathered and its surface is made of coarse grains including some reddish ones probably made of iron oxyde. More posteriorly, it is still partially covered by the dorsal cuticle. The tract has been broken at the level of thoracic segment 10, and the portion of it immediately in front has apparently shrunken a little forwards. A rather deep, broad, sagittal depression is visible on the most anterior portion of the tract. This latter is wide [c. a fourth of the width (tr.) of the occipital ring] and remains so under the glabella. It gently narrows thereafter to represent no more than an eight of the width (tr.) of the axial ring of thoracic segment 9. 3 pairs of digestive caeca occur under the glabella. The two most anterior ones are wide (exs.) but short (tr.) and are obviously partially abraded. The first pair extends abaxially into the shoulders of the hypostome. The third pair is located under the occipital ring and is slightly narrower (exs.), but extend further abaxially. Under the axis of the thorax, only axial ring 1 is clearly associated with a pair of digestive caeca.

BPM 1017. The digestive system is preserved under the posterior two-thirds of the glabella and the axial rings of the 7 anteriormost thoracic segments. Two small portions can also be observed under the posterior part of the pygidial axis. The main portion is composed of the tract and 10 pairs of digestive caeca. Anteriorly, the tract bears a short but broad sagittal depression, giving it the aspect of a bilobate structure. It is rather wide [c. 25 percent of the maximal width of the glabella (tr.)] and remains of roughly the same width under the entire glabella. Then, its width gently decreases posteriorly up to the seventh thoracic segment, where it is half the size it is in the cephalic region. The two small portions of the tract visibly under the pygidial axis are narrow (tr.) and devoid of digestive caeca. The digestive caeca decrease in size from front to rear, and they are all of similar shape. They all have a wide insertion (exs.) on the digestive tract, especially the four most anterior ones. These insertions are not only lateral but extend dorso-medially on about a third of the width (tr.) of the tract. Only the anterior halves (exs.) of the digestive caeca project abaxially and more or less anteriorly on a distance equivalent to half the width (tr) of the tract. The digestive caeca belonging to the first pair slightly differ from the others in exhibiting even wider (exs.) insertions on the tract. The surface of the digestive tract is rough in its most anterior part (up to Dc3), but it becomes increasingly smoother rearwards. A rather large but shallow sagittal furrow is visible all along the dorsal side of the portion of the tract flanked laterally with digestive caeca. The material filling the digestive caeca is coarse, especially for anterior pairs, with some reddish deposits of what may be iron oxides between the grains. A similar reddish material also surrounds like a halo some of the digestive caeca, especially in the cephalic region. A dark staining occurs on the doublural cuticle just posterior to the tip of the pygidial axis, which probably represents material exuded from the anus after the death. In addition, a pair of poorly-defined, phosphatized structures is present under the pygidial pleurae between the axis and the pygidial doublure.

High-resolution microradiographs of the specimen show a darker area medially in the anteriormost portion of the tract, which likely represents the superimposition of an esophagus on the dorsal portion of the tract. This esophagus is narrow anteriorly (about a fifth of the width of the corresponding dorsal tract) but slightly widens (tr.) posteriorly. It is short and directed backwards, the mouth opening just in front of the first pair of digestive caeca. A dark area all around the posterior half of the esophagus may represent some material exuded from the mouth after death.

BPM 1018. Fossilized digestive structures are visible under the posterior part of the glabella, the axial rings of thoracic segments 1 to 7, and under the most posterior part of the pygidial axis. Except for the small posterior portion, the preservation is coarse. The phosphatised digestive structures merge with the dorsal cuticle under the posterior glabella. In front of them is a large area of reddish material, which proved to be non-phosphatised, but particularly rich in carbon (EDX). We interpret it as the remains of the gut content after the gut wall has collapsed. The absence of phosphatisation in this portion of the gut evidences the role of the gut wall in enclosing a microenvironment where conditions conducive for CaPO_4_ precipitation can developed. 6 pairs of digestive caeca, progressively decreasing in size rearwards, can be rather well-individualized from a rather wide digestive tract [*c.* a third of the maximal width (tr.) of the thoracic axis] under the axis in the anterior trunk region. A small portion of the pygidial doublure in the immediate vicinity of the posterior tip of the pygidial axis differs from the surrounding cuticle in being slightly swollen and in having a coarser surface and a darker coloration (enriched in carbon as revealed by EDX). This is most likely the result of the exudation of some material from the anus after death.

BPM 1020. The almost entire digestive system is apparently preserved under the posterior three-fourths of the glabella and the post-cephalic axis up to the posterior tip of the pygidium. However, it is still largely covered by the dorsal cuticle under the glabella and the anterior portion of the thorax up to the fifth thoracic segment, where its presence is mainly evidenced by a darker colour of the cuticle. The distribution of these darker areas suggests the presence of digestive caeca under most of the segments composing this portion of the body, but a precise description of them is not possible. The occurrence of a pair of digestive caeca under the axial ring of the sixth and seventh thoracic segments is also suggested by the rather important width (tr.) of the phosphatised structure. Posteriorly, the tract is narrow [less than a tenth of the width (tr.) of the axis] and devoid of any digestive caeca. It slightly narrows (tr.) rearwards until reaching the posterior tip of the pygidial axis. Just posterior to it, the doublure is darker, suggesting the exudation of material from the anus after the death.

*Coosella kieri* Robison & Babcock, 2011

BPM 1002. A partial preservation of the digestive system can be observed under the posterior third (sag.) of the glabella and the anterior half (sag.) of the thorax. The tract is split medially along the posterior two-thirds of the fossilized portion of the digestive system. This latter is therefore mainly composed of digestive caeca, which are 10 in number. Under the axial ring of thoracic segment 3, the digestive system is broken into an anterior part, apparently not displaced, and a posterior part, which seems to have shrunken along the antero-posterior axis. As a consequence, the axial rings of thoracic segments 3 and 4 are associated with one pair of digestive caeca each, that are slightly displaced backwards, but two pairs of digestive caeca occur under the axial rings of both thoracic segments 5 and 6. Except for the most anterior pair, which is located just posterior to the posterior margin of the hypostome, the digestive caeca project postero-laterally, instead of antero-laterally as in all know examples of preserved digestive caeca of trilobites. They clearly arise in great part from the dorsal side of the tract and they decrease moderately in size from front to rear.

*Genevievella granulatus* (Walcott, 1916)

UU 11071.01. Well-preserved digestive structures are present under the posterior half of the preoccipital glabella. They are masked by the wide (sag.) occipital ring, but a short portion of the tract reappears behind it under the axial ring of thoracic segment 2. The digestive tract is particularly wide anteriorly [c. a fifth of the maximal width (tr.) of the glabella] and it remains so all along the portion visible under the glabella. The small portion located behind the occipital ring is about three times narrower (tr.). A short (sag.) but broad (tr.) sagittal depression is present on the digestive tract anteriorly. Otherwise, the tract is particularly inflated dorsally and there is no clear evidence of a dorsal sagittal furrow. Its surface is particularly uneven and exhibits crystalline inclusions (silica) of about 500 µm. 3 pairs of digestive caeca strongly decreasing in size from front to rear are present under the glabella. The caeca composing the first pair exhibit particularly large insertions (exs.) along the sides of the tract. Their abaxial projections are bent backwards. The digestive caeca of the second pair are also rather widely (exs.) inserted on the tract, but they project antero-laterally. The third pair is composed of more slender caeca, with narrow (exs.) insertions on the tract, which project antero-laterally. No ventral portion of the tract (i.e. oesophagus and mouth) could be observed on microradiographs of the specimen.
