# Supplementary material for: Controls on Gut Phosphatisation: The Trilobites from the Weeks Formation Lagerstätte (Cambrian; Utah)
Source: PLoS One. 2012 Mar 14;7(3):e32934. doi: 10.1371/journal.pone.0032934 (PMC3303877; doi:10.1371/journal.pone.0032934)
Supplement: Table S1 — Energy Dispersive X-ray (EDX) analyses of the digestive structures, cuticle, and matrix in four trilobite specimens from the Middle Cambrian Weeks Formation. Meniscopsia beebei (BPM 1000, BPM 1018, BPM 1020) and Coosella kieri (BPM 1002). Composition is expressed in atomic percentage (%) and the analysed area in square micrometres (µm2). Some elements were not detectable on spectra and therefore were not considered (NC) in the composition estimations. Though recognized on spectra, other elements had estimated proportions below the equipment's lower limit of reliability (0.3%). The digestive structures are characterized by the abundance of P and significantly higher proportion of Ca. The cuticle exhibits the highest proportion of Mg, which is possibly linked to its original composition. The matrix is distinguished from the other two materials by its higher concentration of Al and (to a lesser extent) Fe, as well as the presence of K just above the reliability threshold. The presence of Si in all three materials is probably linked to secondary silicification of the cuticle and (to a lesser extent) the digestive structures, whereas the presence of Si in the matrix is likely the result of aluminosilicate minerals. (DOC) [file pone.0032934.s002.doc]

| **Digestive structures** | | | | | | | | | | | |
| --- | --- | --- | --- | --- | --- | --- | --- | --- | --- | --- | --- |
| **Specimen** | **Area** | **C** | **O** | **F** | **Mg** | **Al** | **Si** | **P** | **K** | **Ca** | **Fe** |
| BPM1000 | 50400 | 25.41 | 56.34 | NC | 0.58 | 0.40 | 1.23 | 5.72 | <0.3 | 9.90 | <0.3 |
| BPM1000 | 28500 | 30.77 | 50.03 | NC | 0.77 | 1.11 | 3.98 | 3.03 | <0.3 | 9.34 | 0.51 |
| BPM1000 | 36100 | 27.54 | 53.97 | NC | 0.68 | 0.52 | 2.20 | 5.34 | <0.3 | 9.28 | <0.3 |
| BPM1000 | 18200 | 17.67 | 56.76 | 1.61 | 0.73 | 0.48 | 3.54 | 4.69 | <0.3 | 13.86 | 0.41 |
| BPM1000 | 2000 | 17.39 | 59.92 | 1.06 | 0.68 | 0.56 | 2.83 | 2.72 | <0.3 | 14.11 | 0.53 |
| BPM1000 | 78300 | 20.76 | 58.25 | 2.13 | 0.68 | <0.3 | 2.26 | 3.65 | <0.3 | 11.59 | <0.3 |
| BPM1000 | 2000 | 13.86 | 57.77 | 4.46 | 0.81 | <0.3 | 1.59 | 7.67 | <0.3 | 13.21 | <0.3 |
| BPM1002 | 15300 | 12.39 | 60.53 | 2.14 | 0.68 | 0.77 | 8.38 | 5.17 | <0.3 | 8.95 | 0.67 |
| BPM1002 | 3600 | 12.14 | 61.11 | 2.30 | 0.67 | 0.55 | 7.96 | 5.28 | <0.3 | 9.17 | 0.56 |
| BPM1002 | 6600 | 14.12 | 59.02 | 2.65 | 0.66 | 0.59 | 7.12 | 5.47 | <0.3 | 9.55 | 0.53 |
| BPM1002 | 48000 | 14.29 | 59.13 | 1.12 | 0.78 | 0.87 | 9.35 | 4.44 | <0.3 | 8.28 | 1.38 |
| BPM1002 | 45000 | 17.13 | 57.40 | 1.61 | 0.72 | 0.78 | 8.99 | 4.33 | <0.3 | 7.80 | 1.00 |
| BPM1002 | 6400 | 22.84 | 56.06 | 1.32 | 0.69 | 0.72 | 6.27 | 3.80 | <0.3 | 7.65 | 0.35 |
| BPM1018 | 12600 | 16.49 | 61.65 | NC | 0.74 | 1.28 | 3.31 | 4.47 | <0.3 | 10.69 | 1.21 |
| BPM1018 | 12100 | 14.64 | 60.81 | NC | 0.72 | 0.73 | 4.52 | 4.79 | <0.3 | 12.68 | 0.80 |
| BPM1020 | 2500 | 24.52 | 52.81 | NC | 0.66 | 0.68 | 6.66 | 3.69 | <0.3 | 10.15 | 0.51 |
| BPM1020 | 2400 | 25.42 | 52.15 | NC | 0.58 | 0.52 | 3.94 | 5.02 | <0.3 | 11.91 | 0.30 |
| BPM1020 | 12000 | 24.67 | 53.00 | NC | 0.52 | 0.46 | 2.42 | 6.90 | <0.3 | 11.57 | 0.30 |
|  | Mean | 19.56 | 57.04 | **2.04** | 0.69 | 0.69 | 4.81 | **4.79** | – | **10.54** | 0.55 |
|  | Min. | 12.14 | 50.03 | 1.06 | 0.52 | 0.40 | 1.23 | 2.72 | – | 7.65 | 0.15 |
|  | Max. | 30.77 | 61.65 | 4.46 | 0.81 | 1.28 | 9.35 | 7.67 | – | 14.11 | 1.38 |
|  | | | | | | | | | | | |
| **Cuticle** | | | | | | | | | | | |
| **Al** | **Area** | **C** | **O** | **F** | **Mg** | **Al** | **Si** | **P** | **K** | **Ca** | **Fe** |
| BPM1000 | 25500 | 18.34 | 57.52 | NC | 1.82 | 0.55 | 17.24 | <0.3 | <0.3 | 4.07 | <0.3 |
| BPM1000 | 72000 | 20.78 | 59.40 | NC | 1.27 | 0.51 | 11.66 | <0.3 | <0.3 | 6.04 | <0.3 |
| BPM1000 | 1600 | 20.14 | 61.83 | NC | 0.95 | 0.78 | 5.58 | <0.3 | <0.3 | 10.21 | <0.3 |
| BPM1000 | 3000 | 22.23 | 56.09 | NC | 1.70 | 0.48 | 14.97 | <0.3 | <0.3 | 4.20 | <0.3 |
| BPM1002 | 19500 | 16.89 | 60.70 | <0.3 | 0.83 | 0.83 | 18.52 | <0.3 | <0.3 | 1.76 | <0.3 |
| BPM1002 | 7200 | 20.68 | 57.55 | <0.3 | 1.01 | 0.70 | 17.08 | <0.3 | <0.3 | 2.56 | <0.3 |
| BPM1002 | 72800 | 19.05 | 59.41 | <0.3 | 0.86 | 0.76 | 17.51 | <0.3 | <0.3 | 2.11 | <0.3 |
| BPM1002 | 49400 | 18.21 | 60.28 | <0.3 | 0.86 | 0.69 | 18.58 | <0.3 | <0.3 | 1.37 | <0.3 |
| BPM1002 | 27200 | 13.67 | 62.66 | <0.3 | 0.84 | 0.59 | 20.53 | <0.3 | <0.3 | 1.52 | <0.3 |
| BPM1018 | 59800 | 21.87 | 57.48 | NC | 1.88 | 0.39 | 15.28 | <0.3 | <0.3 | 2.81 | <0.3 |
| BPM1018 | 64800 | 21.18 | 58.51 | NC | 1.79 | 0.30 | 15.14 | <0.3 | <0.3 | 2.80 | <0.3 |
| BPM1018 | 58800 | 20.79 | 56.84 | NC | 1.56 | 0.45 | 16.71 | <0.3 | <0.3 | 3.35 | <0.3 |
| BPM1018 | 24000 | 17.20 | 59.74 | NC | 1.06 | 0.92 | 17.26 | <0.3 | <0.3 | 2.47 | 0.94 |
| BPM1018 | 8000 | 14.98 | 60.76 | NC | 0.91 | 0.57 | 19.80 | <0.3 | <0.3 | 2.15 | 0.49 |
| BPM1018 | 210700 | 15.52 | 60.49 | NC | 0.88 | 1.16 | 17.28 | <0.3 | <0.3 | 3.55 | 0.60 |
| BPM1020 | 95000 | 25.24 | 54.94 | NC | 1.63 | 0.40 | 14.29 | <0.3 | <0.3 | 3.17 | <0.3 |
| BPM1020 | 42000 | 30.26 | 52.14 | NC | 1.43 | 0.48 | 12.71 | <0.3 | <0.3 | 2.75 | <0.3 |
|  | Mean | 19.83 | 58.61 | – | **1.25** | 0.62 | 15.89 | – | – | 3.35 | 0.68 |
|  | Min. | 13.67 | 52.14 | – | 0.83 | 0.30 | 5.58 | – | – | 1.37 | 0.49 |
|  | Max. | 30.26 | 62.66 | – | 1.88 | 1.16 | 20.53 | – | – | 10.21 | 0.94 |
|  | | | | | | | | | | | |
| **Matrix** | | | | | | | | | | | |
| **Specimen** | **Area** | **C** | **O** | **F** | **Mg** | **Al** | **Si** | **P** | **K** | **Ca** | **Fe** |
| BPM1000 | 31500 | 17.57 | 60.02 | NC | 1.13 | 4.14 | 12.67 | <0.3 | 0.96 | 2.11 | 0.99 |
| BPM1000 | 10400 | 24.86 | 53.10 | NC | 1.03 | 4.54 | 12.30 | <0.3 | 1.13 | 1.57 | 1.04 |
| BPM1000 | 78400 | 12.40 | 64.05 | NC | 1.38 | 3.75 | 11.89 | <0.3 | 0.82 | 4.57 | 0.91 |
| BPM1000 | 25600 | 13.45 | 63.61 | NC | 1.55 | 3.23 | 12.12 | <0.3 | 0.72 | 4.26 | 0.95 |
| BPM1000 | 8000 | 17.85 | 59.61 | NC | 1.30 | 3.34 | 11.61 | <0.3 | 0.79 | 4.44 | 0.92 |
| BPM1002 | 40000 | 17.13 | 61.31 | <0.3 | 0.83 | 2.95 | 9.91 | <0.3 | 0.67 | 6.65 | 0.72 |
| BPM1002 | 40000 | 17.33 | 60.92 | <0.3 | 0.74 | 2.78 | 10.07 | <0.3 | 0.67 | 6.84 | 0.69 |
| BPM1018 | 36000 | 13.68 | 60.66 | NC | 1.15 | 5.42 | 11.00 | <0.3 | 1.58 | 4.52 | 1.57 |
| BPM1020 | 57600 | 16.64 | 60.48 | NC | 0.93 | 3.22 | 11.51 | <0.3 | 0.82 | 4.91 | 1.27 |
| BPM1020 | 30600 | 20.30 | 59.23 | NC | 0.78 | 2.57 | 9.54 | <0.3 | 0.62 | 5.97 | 0.82 |
| BPM1020 | 34200 | 18.68 | 60.23 | NC | 0.67 | 2.36 | 9.90 | <0.3 | 0.52 | 6.63 | 0.91 |
|  | Mean | 17.26 | 60.29 | – | 1.04 | **3.48** | 11.14 | – | **0.85** | 4.77 | **0.98** |
|  | Min. | 12.40 | 53.10 | – | 0.67 | 2.36 | 9.54 | – | 0.52 | 1.57 | 0.69 |
|  | Max. | 24.86 | 64.05 | – | 1.55 | 5.42 | 12.67 | – | 1.58 | 6.84 | 1.57 |
|  | | | | | | | | | | | |
