# Supplementary material for: Controls on Gut Phosphatisation: The Trilobites from the Weeks Formation Lagerstätte (Cambrian; Utah)
Source: PLoS One. 2012 Mar 14;7(3):e32934. doi: 10.1371/journal.pone.0032934 (PMC3303877; doi:10.1371/journal.pone.0032934)
Supplement: Table S2 — Mass spectrometry analyses of the matrix of BPM 1001 (Meniscopsia beebei) from the Middle Cambrian Weeks Formation. Concentrations are expressed in parts per million (ppm). Note the extremely low value for P. (DOC) [file pone.0032934.s003.doc]

| **BPM 1001 – Matrix (ppm)** | | | | | | | | | | |
| --- | --- | --- | --- | --- | --- | --- | --- | --- | --- | --- |
|  | **^23^Na** | **^24^Mg** | **^27^Al** | **^31^P** | **^34^S** | **^39^K** | **^43^Ca** | **^47^Ti** | **^55^Mn** | **^56^Fe** |
| Sample 1 | 693.5 | 7598.8 | 47410.7 | **288.5** | 0.00 | 16980.9 | 184562.6 | 3448.6 | 259.3 | 22578.4 |
| Sample 2 | 659.8 | 7311.2 | 44877.9 | **284.0** | 0.00 | 16707.6 | 178909.2 | 3378.1 | 255.0 | 22269.5 |
